# Disturbance theory for ecosystem ecologists: A primer

**DOI:** 10.1002/ece3.11403

**Published:** 2024-05-30

**Authors:** Christopher M. Gough, Brian Buma, Anke Jentsch, Kayla C. Mathes, Robert T. Fahey

**Affiliations:** ^1^ Department of Biology, College of Humanities & Sciences Virginia Commonwealth University Richmond Virginia USA; ^2^ Environmental Defense Fund Boulder Colorado USA; ^3^ Department of Integrative Biology University of Colorado Denver Denver Colorado USA; ^4^ Department of Disturbance Ecology and Vegetation Dynamics, Bayreuth Center of Ecology and Environmental Research (BayCEER) University of Bayreuth Bayreuth Germany; ^5^ Department of Natural Resources and the Environment & Center for Environmental Sciences and Engineering University of Connecticut Storrs Connecticut USA

**Keywords:** disturbance ecology, ecological theory, ecosystem ecology, ecosystem functioning, net primary production, resilience, resistance, stability, succession, system dynamics

## Abstract

Understanding what regulates ecosystem functional responses to disturbance is essential in this era of global change. However, many pioneering and still influential disturbance‐related theorie proposed by ecosystem ecologists were developed prior to rapid global change, and before tools and metrics were available to test them. In light of new knowledge and conceptual advances across biological disciplines, we present four disturbance ecology concepts that are particularly relevant to ecosystem ecologists new to the field: (a) the directionality of ecosystem functional response to disturbance; (b) functional thresholds; (c) disturbance–succession interactions; and (d) diversity‐functional stability relationships. We discuss how knowledge, theory, and terminology developed by several biological disciplines, when integrated, can enhance how ecosystem ecologists analyze and interpret functional responses to disturbance. For example, when interpreting thresholds and disturbance–succession interactions, ecosystem ecologists should consider concurrent biotic regime change, non‐linearity, and multiple response pathways, typically the theoretical and analytical domain of population and community ecologists. Similarly, the interpretation of ecosystem functional responses to disturbance requires analytical approaches that recognize disturbance can promote, inhibit, or fundamentally change ecosystem functions. We suggest that truly integrative approaches and knowledge are essential to advancing ecosystem functional responses to disturbance.

## Graphical abstract

Disturbance is a central theme in ecosystem ecology. Equal measures viewpoint and review, our article concisely highlights and recommends how the concepts and findings of other biological disciplines can enrich the understanding and interpretation of how disturbance affects ecosystem processes.

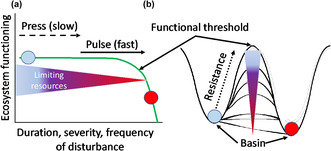

## 1 INTRODUCTION

Disturbances affect every scale and level of biological organization. However, disturbance studies are generally guided by discipline‐specific theories, terminology, and literature, limiting coherence across fields of ecology. In ecosystem ecology, prominent historical and enduring theoretical frameworks emphasize disturbance effects on systems‐level biomass, and energy pools and fluxes over time and space (Bormann & Likens, 1979; Odum, 1969; Whittaker et al., 1974). While the influence of these theories continues, their inception did not account for interactions with rapidly changing climate or climate extremes, permanent (i.e., state‐) changes in biogeochemical cycles, species introductions, or novel disturbances (Corman et al., 2019; Sala et al., 2000). Yet, the multitemporal and spatially integrative nature of ecosystem ecology requires long‐term consideration (Gaiser et al., 2020) of uncertain future conditions (Stern, 2008), dynamic resource ratios and stoichiometries (Jentsch & White, 2019), and community‐to‐landscape structural reorganization (Carpenter et al., 2001; Pickett et al., 2011; Scheffer et al., 2001; Scheffer & Carpenter, 2003). Moreover, many historical conceptual models still embraced by ecosystem ecologists were not testable when proposed because of technological constraints and more limited quantitative metrics and methods. For example, Odum's (1969) seminal work, “The Strategy of Ecosystem Development,” which is cited more now than it was a half century ago, long‐preceded meteorological “flux” tower networks (Baldocchi, 2008; Novick et al., 2018) measuring ecosystem processes such as net ecosystem CO_2_ exchange and ecosystem respiration, nomenclature that was standardized in the 21st Century (Chapin et al., 2006).

Now, following decades of observations and theoretical advances (Gaiser et al., 2020; Jentsch & White, 2019; Kranabetter et al., 2016; Lin et al., 2022), we consider how contemporary disturbance theory and knowledge can inform core themes addressed by ecosystem ecologists. Here, the term *functioning* encompasses system‐wide processes, such as net primary production, ecosystem respiration, evapotranspiration, and energy balance. Rather than an exhaustive review, we present a broadly accessible outline for the novice in advance of a more comprehensive dive into a rich but technical literature spanning multiple biological disciplines and decades. We conclude by inviting readers to contribute their own commentary and suggested readings, acknowledging that interdisciplinary perspectives, theories, and observations are necessary to enrich and unify disturbance ecology paradigms.

## 2 DISTURBANCE MAY STIMULATE, REDUCE, OR CREATE NEW ECOSYSTEM FUNCTIONS, ALL AT THE SAME TIME

With the origins of disturbance theory rooted outside of ecosystem ecology (e.g., Clements, 1916; Gleason, 1917), conceptual and analytical frameworks for interpreting disturbance responses historically emphasized population, community, and, later, landscape structure (Pickett et al., 2011) rather than ecosystem function (Pulsford et al., 2016). For example, White and Pickett's (1985) often‐cited definition describes disturbance as a discrete event in time and space “that disrupts the *structure* of an ecosystem, community, or population, and changes resource availability or the physical environment.” While this definition does not exclude ecosystem functioning, its emphasis underscores foundations outside of ecosystem ecology.

With an emphasis on disruptive effects, many conceptual and analytical models adopted by ecosystem ecologists assume or predict a loss of ecosystem functioning following disturbance relative to a control or baseline (Amiro et al., 2010; Anderegg et al., 2016). In nature, however, different disturbances have different impacts on ecosystem processes, and different functions may have different responses to the same disturbance. For example, disturbance severity has variable effects on processes regulating forest carbon uptake and loss (Clay et al., 2022; Gough, Atkins, et al., 2021; Shabaga et al., 2022). Over successional timescales, disturbance may stimulate some functions at the expense of others, for example, by increasing nitrogen leaching and decreasing nitrogen‐limited primary production (White et al., 2004). While disturbances sometimes reduce the population sizes of dominant species and drastically alter community structure (e.g., by reducing biodiversity, Hillebrand & Kunze, 2020), the reallocation of limiting resources such as light, nutrients, and water may also increase whole‐ecosystem resource‐use efficiency. For example, phloem‐disrupting disturbances that killed a fraction of trees and reduced species richness increased carbon‐use efficiency and, consequently, enhanced the primary production of a temperate forest (Gough, Bohrer, et al., 2021). Moderate severity or partial disturbances from fire, wind, or thinning that reduce competition and liberate growth‐limiting resources can similarly increase the production of temperate and tropical forests (Buma & Schultz, 2020; Kweon & Comeau, 2019; Munoz et al., 2021; Nunes et al., 2018). Thus, there is no consistent impact of disturbance on the (positive, negative, or neutral) directionality of ecosystem functioning and, in some cases, opposing processes offset one another, limiting the “net effects” (sensu Pickett et al., 2009) of disturbance on integrative ecosystem processes. For example, low intensity disturbances that reduce plant competition may increase ecosystem carbon uptake (i.e., gross primary production, GPP) and carbon losses (i.e., ecosystem respiration, ER), resulting in no change in net ecosystem CO_2_ exchange (=GPP–ER) (Gough, Bohrer, et al., 2021).

**FIGURE 1:**
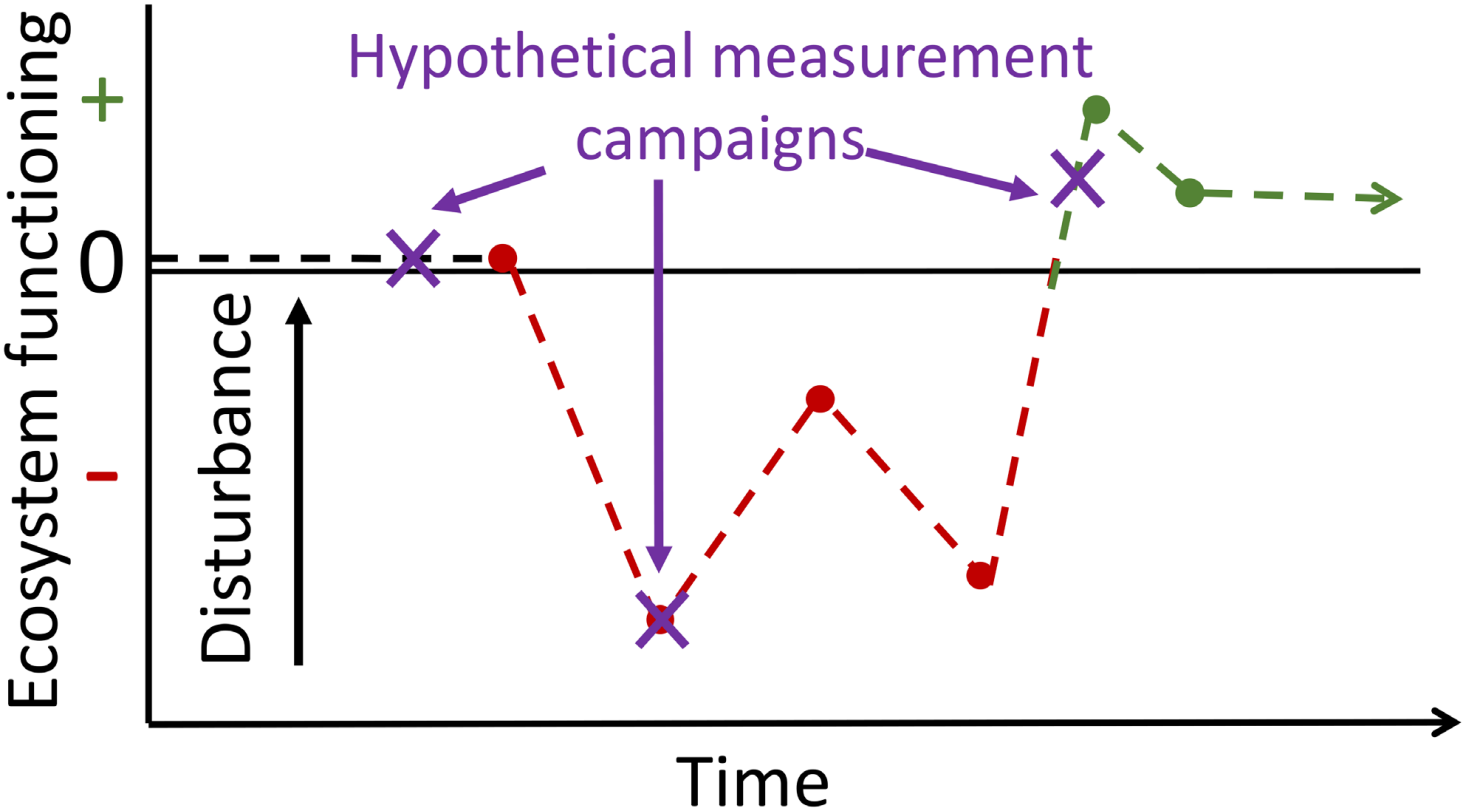
Disturbance is often assumed to have disruptive effects on ecosystem processes, but observed responses may be positive, negative, or neutral and change over time, influencing how disturbance effects are perceived and reported. Such dynamic responses may also explain why conflicting disturbance responses are reported in the literature and underscore the need for long‐term repeated measurements.

Analytical frameworks that accommodate the multiple directional and temporal responses of ecosystem functioning to disturbance have been proposed and are described in detail elsewhere (Figure 1; Mathes et al., 2021). These frameworks help differentiate and interpret time‐dependent disturbance responses, and highlight how the apparent effects of disturbance on ecosystem functioning depends on when measurements are taken. While not yet widely embraced by ecosystem ecologists, the use of such frameworks could help address a number of knowledge gaps in the realm of disturbance ecology, including three fundamental aspects: First, to what extent structure and function are coupled following disturbance; second, whether initial responses to disturbance predict long‐term change; and third, which disturbance regimes and sources deplete versus enhance structure and function.

## 3 NON‐LINEAR THRESHOLD RESPONSES TO DISTURBANCE ARE COMMON

Ecosystem ecologists have long‐considered how ecosystem functions respond to disturbance. For example, the effects of different disturbance sources (e.g., fire, insects, wind) on systems‐level carbon cycling processes have been examined in several ecosystems (Amiro et al., 2010; Rebane et al., 2019; Senf & Seidl, 2021). Theory, experiments, and models generally assume that for most functional processes, the magnitude of change is correlated with disturbance frequency, severity, or duration (Anderegg et al., 2015; Bond‐Lamberty et al., 2015). For example, insects killing 50% of all trees within a forest stand are expected to reduce net primary production by a similar amount, a logical hypothesis that is sometimes observed in nature (Hicke et al., 2012) and routinely predicted by models (Bond‐Lamberty et al., 2015). Although predicting thresholds is difficult (Hillebrand et al., 2020), some ecosystems absorb substantial disturbance without commensurate changes in functioning, exhibiting non‐linear *threshold* responses to more frequent, severe, or longer lasting disturbances (Flower & Gonzalez‐Meler, 2015; Stuart‐Haentjens et al., 2015). Indeed, non‐linear changes in ecosystem composition and structure are increasingly reported, motivating novel research questions asking why ecosystem processes respond with varying degrees of resilience to disturbance (Turner et al., 2020).

The concept of non‐linear thresholds and the statistical tools for their detection (Jiang et al., 2018; Lenton et al., 2008, 2019; Toms & Lesperance, 2003) are widespread across ecological disciplines (Briske et al., 2005; Groffman et al., 2006; Johnston et al., 2021), but underutilized by ecosystem ecologists. Ecological thresholds include non‐linear changes in populations, community and landscape structure, and ecosystem processes following disturbance (Groffman et al., 2006) such as changing resource ratios and limiting factors (Jentsch & White, 2019), and their detection, description, and overall typology depends heavily on the organizational scale being observed (Spake et al., 2022). The published literature contains relatively few studies emphasizing ecosystem‐scale functioning. For example, a *Web of Science* key word search (on 03‐08‐24) yielded 414 articles referencing “threshold*” and “ecolog*” and “ecosystem function*”, while substituting the latter for “communit*” and “population*” returned 3865 and 4868 articles, respectively. Moreover, population and community—rather than ecosystem—ecologists have generally led advances in the conceptualization of ecological thresholds, including the data visualization and quantification of non‐linear behavior (Jentsch & White, 2019; Seidl et al., 2016) and the application of basin attractor analogies (Holling, 1973; Huisman & Weissing, 2001; van Nes & Scheffer, 2007).

**FIGURE 2:**
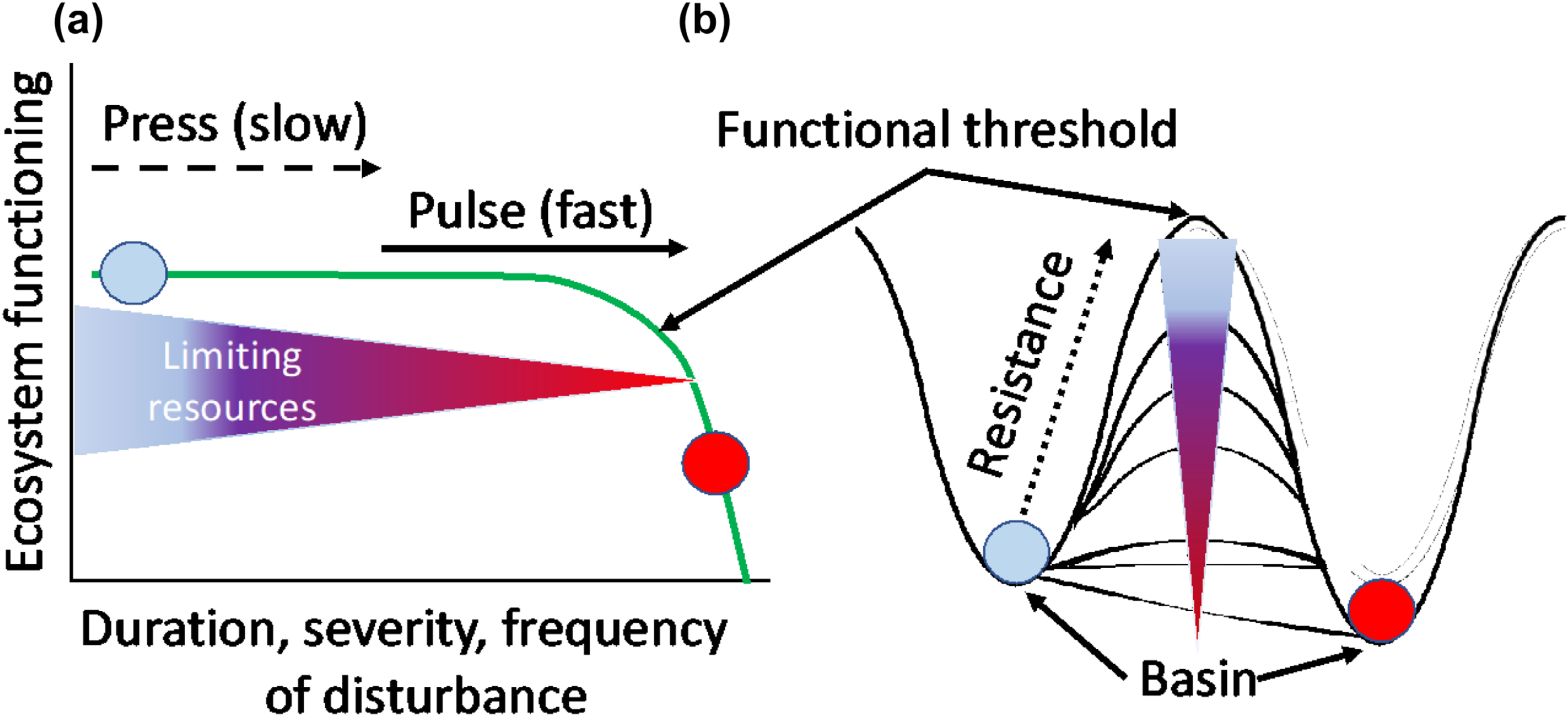
Drawing from theoretical frameworks developed by community ecologists and recent observations of ecosystem processes, functional thresholds can be conceptualized as abrupt non‐linear transitions from one functional regime to another resulting from press or pulse disturbance (a) and using a basin attractor analogy (b). Press disturbances, such as sustained drought or gradually rising temperatures, may push a function closer to its threshold as limiting resources decline, priming the system for greater sensitivity to subsequent pulse disturbance.

When integrated with ecosystem ecology principles, population‐, community‐, and landscape ecology‐originated theories provide a basis for interpreting the mechanisms underlying ecosystems' response to disturbance. For example, disturbance has non‐random impacts that are dependent upon frequency, severity, source, and duration, resulting in the retention of different biotic (e.g., species abundances) and abiotic (e.g., nutrient capital) legacies. Impacted ecosystems may maintain pre‐disturbance functioning, but are frequently more fragile as a result, leading to threshold behavior if additional stressors or interacting, compound disturbances occur (Burton et al., 2020; Johnstone et al., 2016; Peterson, 2019). Similarly, slow and lasting “press” disturbances such as prolonged drought may incrementally exhaust material legacies at broad scales (Smith et al., 2024) until a more abrupt “pulse” disturbance, like extreme weather events or insect mortality, pushes the system beyond its limit, resulting in threshold change and potential reorganization that forces new stable dynamics (Harley & Paine, 2009; Renwick et al., 2016). Merging these concepts, thresholds in ecosystem functioning can be illustrated as a basin attractor model, in which a loss of limiting resources or material legacies linearly or non‐linearly diminishes functioning, and reduces the barrier to permanent functional regime change (Figure 2).

## 4 DISTURBANCE GIVES RISE TO MULTIPLE SUCCESSIONAL PATHWAYS

The interplay between ecological succession and disturbance has been an object of theoretical and empirical study for over a century (Shelford, 1912), with ecosystem ecologists considering functioning in this context by the middle 20th century (Odum, 1969; Whittaker, 1960). Initial theoretical models and observations emphasized a single axis of successional change, with disturbance partially or fully resetting succession, depending on the degree of severity (e.g., Tansley, 1935, Figure 2a). Some conceptions were dominated by primarily a single trajectory, while others allowed for alternative trajectories (Connell & Slatyer, 1977) depending on initial conditions, but disturbance still played a “resetting” role (Young et al., 2001). In this general model, primary production increases rapidly in young, aggrading ecosystems as pioneer plant species with little competition and an abundance of resources populate an area and grow rapidly; eventually, primary production stabilizes as mortality and replacement achieve steady state. In some ecosystems, arrested succession (Walker & del Moral, 2003) or retrogression emerges as declines in nutrient availability or other constraints begin to limit productivity (Peltzer et al., 2010). With recognition that there are exceptions to this general trajectory (Pulsford et al., 2016), observations show that primary production, in the absence of disturbance, aligns with early theory and progresses over timescales of decades to centuries in a relatively predictable and conserved way (Luyssaert et al., 2008; Pregitzer & Euskirchen, 2004).

Early theorists and empiricists, however, generally formulated their understanding in the absence of novel disturbance regimes and rapid climate change and without the benefit of modern ecosystem‐scale measurements of biomass pools and fluxes. Moreover, they typically assumed that disturbance categorically reset—partially or fully—ecosystem functioning (Grime, 1979). While contemporary disturbance theory allows for multiple successional pathways (Pickett et al., 2009), such flexibility is not often represented in popular conceptions of succession–disturbance interactions, including foundational ecology texts. Indeed, a Google search (10‐19‐22) of “ecological succession” and “ecological succession and disturbance” yielded only textbook illustrations of linear, single‐axis change, and, when depicted, disturbance without exception rewound the successional clock (Figure S1).

Outside of ecosystem ecology, examples of “accelerated” succession and even full ecological regime change abound and inform a more nuanced model of how succession–disturbance interactions influence functioning (Higgs et al., 2018). For example, moderate severity disturbances causing only partial mortality can promote microclimatic conditions that favor shade‐tolerant late successional, rather than pioneer, species (Abrams & Scott, 1989; Fahey et al., 2015; Jenkins & Parker, 1998; Meigs & Keeton, 2018; Trammell et al., 2017). Severe or frequent (Calder & Shuman, 2017; Johnstone et al., 2020), linked or compounding (Buma, 2015; Crausbay et al., 2017), or novel disturbances (Dijkstra et al., 2017) can redirect community successional dynamics altogether into new regimes, giving rise to separate axes of functional change and, possibly, long‐term stability (Buma, Harvey, et al., 2019; Jasinski & Payette, 2005; Williams et al., 2011); furthermore, community composition interacts with disturbance history (Averill et al., 2022). Examples of functional regime change at the ecosystem scale (Scheffer et al., 2001), while less documented, include coral reef shifts from coral‐ to algal‐dominated systems, with concurrent changes in productivity and nutrient status (Crisp et al., 2022; Nystrom et al., 2000), shifts between forests and grass dominated systems (Berdugo et al., 2022; Buma & Wessman, 2011), or major changes in hydrological functioning associated with fire in fire‐naive forest ecosystems, leading to waterlogging and subsequent conversion to bog‐like landscapes (Diaz et al., 2007). In some cases, disturbances restructure ecosystems, making them more functionally resistant to emerging climate conditions (Buma & Schultz, 2020; Thom et al., 2017). These examples demonstrate the potential for disturbances to push ecosystems along multiple axes over long timescales—not only the “traditional” forward or backward on a pre‐defined successional continuum but also in alternate and novel directions.

**FIGURE 3:**
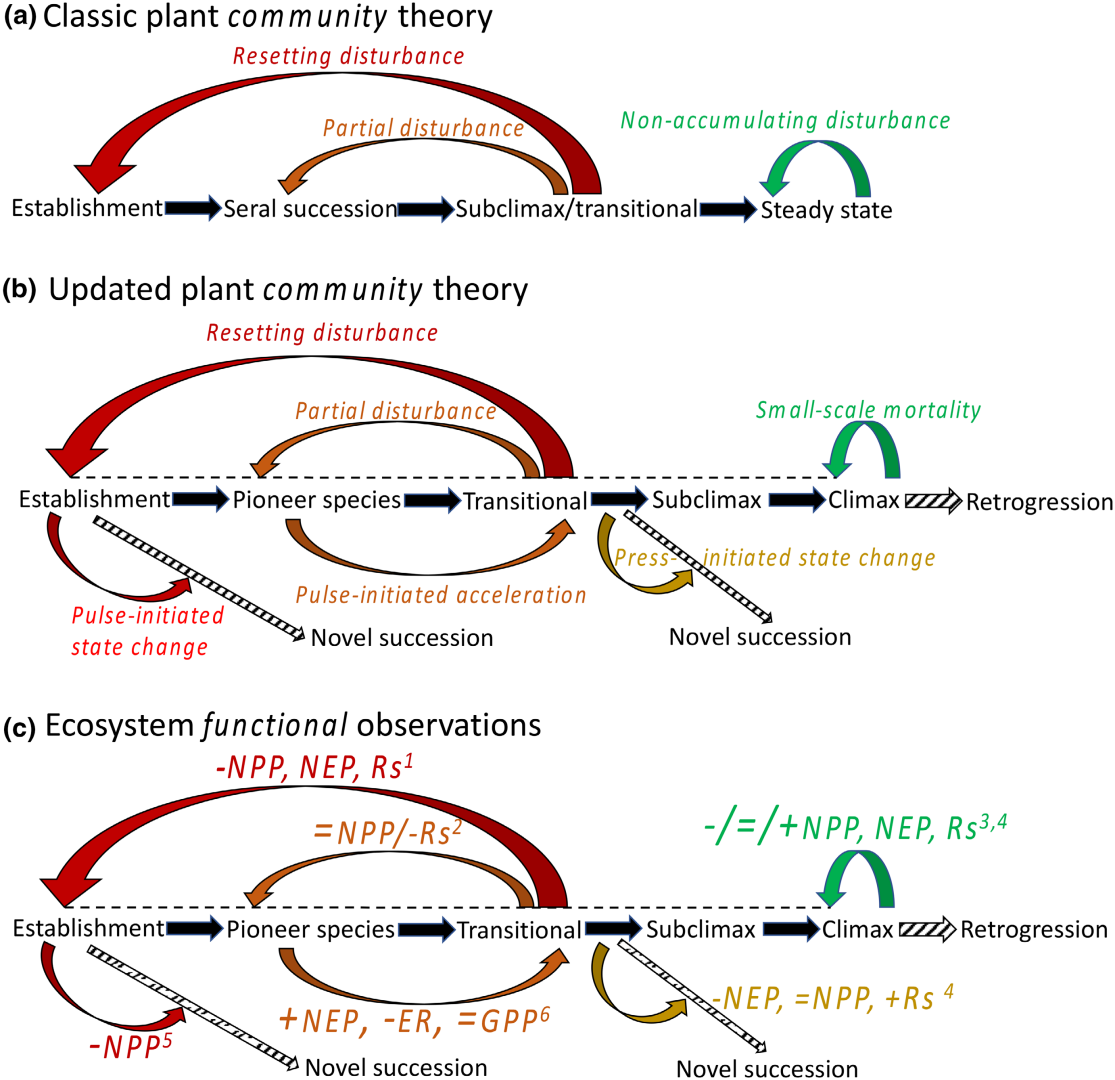
Early community ecology‐driven successional theory posited and often observed partial to full resetting of plant community development in response to disturbance (a). Observations of plant community and ecosystem functioning dynamics suggest that disturbance can alternatively advance or change axes of succession altogether, and site degradation can lead to retrogression. Disturbance may increase some elements of ecosystem functioning, while reducing others, resulting in potentially neutral “net effects” in which opposing fluxes offset one another. For example, within the same north temperate forested landscape, different neighborhood‐scale disturbance–succession interactions caused variable initial responses in net primary production (NPP), net ecosystem production (NEP), soil respiration (Rs), gross primary production (GPP), and ecosystem respiration (ER). ‐, =, and + indicate negative, neutral, and positive responses; ^1^Gough et al. 2007; ^2^Gough et al. 2021; ^3^Scheuermann et al. 2018; ^4^Clay et al. 2022; ^5^Stuart‐Haentjens et al., 2023; ^6^Gough et al. 2021.

We suggest that ecosystem ecology more broadly adopt updated conceptual frameworks that acknowledge disturbances can reset or increase functioning or redirect successional trajectories all together (Pickett et al., 2011). Indeed, rate and direction of successional dynamics after disturbance depend on local energy flux potential, resource availability, and biotic traits (Jentsch & White, 2019). For example, in secondary succession, rates of change are initially high and decrease through time as available resources are accumulated in biomass or are lost from circulation, again highlighting how measurement timing influences the interpretation of disturbance response. While the original model of succession (Figure 3a) may be valid under some conditions, this updated framing is more realistic and opens avenues for steering toward future directions of sustained ecosystem functioning in an era of global change and shifting disturbance regimes (Figure 2b). Moreover, an updated model of functional disturbance–succession interactions should acknowledge the “accelerating” effects of some disturbances, particularly those that reduce or eliminate early successional species and produce greater biological and structural complexity.

## 5 MULTIPLE FORMS OF DIVERSITY SUPPORT FUNCTIONAL STABILITY, MOSTLY FOR THE SAME REASONS

Biological diversity plays a key role in the stabilization of cellular to landscape processes and is therefore a central determinant of disturbance response across scales of biological organization. For example, functionally redundant gene products provide “functional buffering” at the cellular level; response mechanism diversity (Elmqvist et al., 2003) and genetic diversity (Schippers et al., 2015) provide analogous landscape‐scale stability following disturbance (Frelich & Reich, 1999; Kellner et al., 2009; Li et al., 2003; Scholl et al., 2023). While functional redundancy underlies stability across levels of biological organization, scale‐centric biological disciplines sometimes approach, conceptualize, and define diversity differently.

Moreover, while the interplay between structural, genetic, trophic, trait, and other aspects of diversity that give rise to ecosystems' functional redundancy are debated in the literature (Eisenhauer et al., 2019), the controlling variables are tightly intertwined in nature. For example, inter‐ and intraspecific genetic diversity, species diversity, and structural diversity are correlated in forest communities (Gough et al., 2020), suggesting that the isolation of a single controlling influence is impossible in natural (but perhaps not constructed) ecosystems. Attempts to identify the effects of single metrics of diversity on functioning are likely insufficient and may miss important covariates or potentially confound unmeasured causes with measured correlates (Buma, Bisbing, et al., 2019). Moreover, functional trade‐offs determine community and ecosystem responses to disturbance across biomes (Conti et al., 2023). Therefore, we suggest that models and conceptual frameworks considering diversity's effects on ecosystem functioning incorporate a multivariate perspective with input from a variety of disciplines, including molecular biologists focused on genetic diversity, community ecologists emphasizing species diversity, and ecosystem ecologists studying structural diversity and biogeochemical interactions across trophic levels.

## 6 CONCLUSIONS

Disturbances are changing in frequency, intensity, and cause worldwide (e.g., in forests: Weed et al., 2013, Seidl et al., 2017; grasslands: Joyce et al., 2016; Chen et al., 2023; drylands: Maestre et al., 2022; coral: Vercelloni et al., 2020; Chen et al., 2023). In addition to advancing fundamental knowledge in disturbance ecology (Wohlgemuth et al., 2022), updated and more integrative theories relevant to ecosystem functioning are needed to guide disturbance management, and better anticipate and simulate ecosystems' responses to disturbance in this era of rapid global change. The effect of disturbance on ecosystem processes will be a primary determinant of the future functioning and service provisioning of ecosystems in the face of these changing disturbance regimes (Seidl et al., 2016). Understanding the varied impacts of disturbance on ecosystem functions will be an essential component of both recognizing and mitigating the effects of climate and global change factors on the health of ecosystems (Thom et al., 2017). For example, monitoring of ecosystem functions can provide an “early warning system” of potential ecosystem transitions or state changes (Contosta et al., 2023; Keen et al., 2022). The frameworks discussed here highlight the value of integrative theory when considering applications and illustrate a potential roadmap for incorporating multiple response types and trajectories into long‐term ecosystem monitoring practice. In addition to monitoring, ecosystem functional response to disturbance can be used as both a predictor and outcome assessment tool for evaluating the impact of management focused on promoting ecosystem adaptation to climate change and related stressors (Seidl & Turner, 2022). For example, climate‐adaptive management in forested ecosystems is generally conducted using silvicultural plans that focus on forest structure and species composition and diversity (Janowiak et al., 2014; Nagel et al., 2017), but often with the goal of promoting stability in functions such as carbon or water cycling (Halofsky et al., 2018; Ontl et al., 2020). Understanding how disturbance structural outcomes and changes in species identities, traits, and diversity are linked with the response of ecosystem functions is therefore essential to understanding both near‐term responses of forests to climate‐adaptive management and also the longer‐term response of future ecosystems to projected changes (Aquilué et al., 2020; Clark et al., 2022; Messier et al., 2019).

While disturbance occurs at all scales of biological organization, disciplinary science has sometimes resulted in disparate rather than integrative theories, terminology, and concepts. Comprehensively updating disturbance theories relevant to ecosystem ecologists requires outside‐of‐the‐disciplinary‐box thinking, and such thinking necessitates reading, discussion, and research that spans disciplines. While not exhaustive, Table 1 provides a sampling of literature from biological disciplines outside of ecosystem ecology that is relevant to the four theoretical areas discussed in this commentary. We invite your contributions to this list via https://osf.io/a5zvp/.

**TABLE 1:** Disturbance theoretical frameworks originating outside of ecosystem ecology with applicability to ecosystem functioning.

Theory	Origin	What it said:	How it applies to ecosystem ecology:	References
Biogeochemical dynamics	Biogeo‐chemistry	The partitioning ratio of soil and plant nutrient stocks will undergo a predictable trajectory after disturbance.	Offers a framework to assess ecosystem biogeochemical response to disturbance using nutrient partitioning ratios.	Kranabetter et al., (2016)
Multidimensional stability	Population and community ecology	There are multiple, quantifiable dimensions of community and population response to disturbance.	Provides a conceptual and mathematical framework for interpreting and comparing ecosystem functional responses to disturbance.	Hillebrand et al., (2018); Mathes et al., (2021)
Intermediate disturbance hypothesis	Community ecology	Moderate intensity disturbances may increase species diversity by augmenting or diversifying habitat and resource availability.	Species diversity, habitat breadth, and resource availability affect ecosystem functional responses to disturbance, suggesting moderate intensity disturbance could increase mass and energy fluxes.	Huston, (2014)
Disturbance legacies	Population and community ecology	Traits and adaptations, as well as the residual abiotic and biotic materials that persist through disturbance determine ecological responses.	Disturbance legacies may be critical determinants of ecosystem functional responses to disturbance.	Johnstone et al., (2016)
Tipping points, thresholds, and alternate stable states	Population and community ecology	High intensity or frequency disturbance may force a permanent (i.e., stable) shift in population or community structure.	Ecosystems may exhibit similar non‐linear threshold responses to disturbance, changing long‐term functioning.	Scheffer & Carpenter, (2003)
Diversity and resilience	Community ecology	Diverse communities respond to disturbance with greater functional stability.	Diversity, broadly defined, may increase the stability of ecosystem functioning.	Oliver et al., (2015)
Landscape dynamics	Landscape ecology	Spatially and temporally asynchronous disturbance responses, when balanced, may have a stabilizing influence over landscape level structure and function.	Patchy disturbance within an ecosystem may not be functionally destabilizing when uniform in time and space.	Turner, (2010)
Functional buffering	Cellular biology	The functional redundancy of cellular components rescues whole‐cell function.	Functional buffering mechanisms exist across levels of biological organization, from cellular to ecosystems	Lin et al., (2022)
Abrupt Changes in Ecosystems	System theory	Interactions among multiple drivers often produce abrupt change in ecosystems.	Suggests research priorities to advance understanding of abrupt changes in ecosystems in the face of climate change.	Turner et al., (2020)
Net effects and indeterminate directionality of successional processes	Community ecology	Ecological restoration and succession more generally, is informed by synthetic and updated vegetation dynamic theories that consider net effects and indeterminate successional pathways.	Just as the net effects of multiple interacting community processes influence overall vegetation dynamics, ecosystem processes such as *net* primary production and *net* ecosystem production are determine by aggregate and sometimes opposing flux responses.	Pickett et al., (2009)

*Note*: We invite additional recommendations and comments from the community here: https://osf.io/a5zvp/.

## AUTHOR CONTRIBUTIONS

**Christopher M. Gough:** Conceptualization (lead); funding acquisition (lead); project administration (lead); writing – original draft (lead); writing – review and editing (equal). **Brian Buma:** Conceptualization (equal); writing – original draft (equal); writing – review and editing (equal). **Anke Jentsch:** Validation (equal); writing – review and editing (equal). **Kayla C. Mathes:** Writing – review and editing (equal). **Robert T. Fahey:** Validation (equal); writing – review and editing (equal).

## Supporting information


Figure S1.

## Data Availability

No data are reported in this manuscript. Figures are conceptual and not derived from actual data.
