# Supplementary material for: Disturbance theory for ecosystem ecologists: A primer
Source: Ecol Evol. 2024 May 30;14(6):e11403. doi: 10.1002/ece3.11403 (PMC11139967; doi:10.1002/ece3.11403)
Supplement: Supplementary file 1 — Figure S1. [file ECE3-14-e11403-s001.docx]

Figure S1. First-page Google image search returns for keywords “ecological succession” (A) and “ecological succession and disturbance” (B), October 19, 2022.


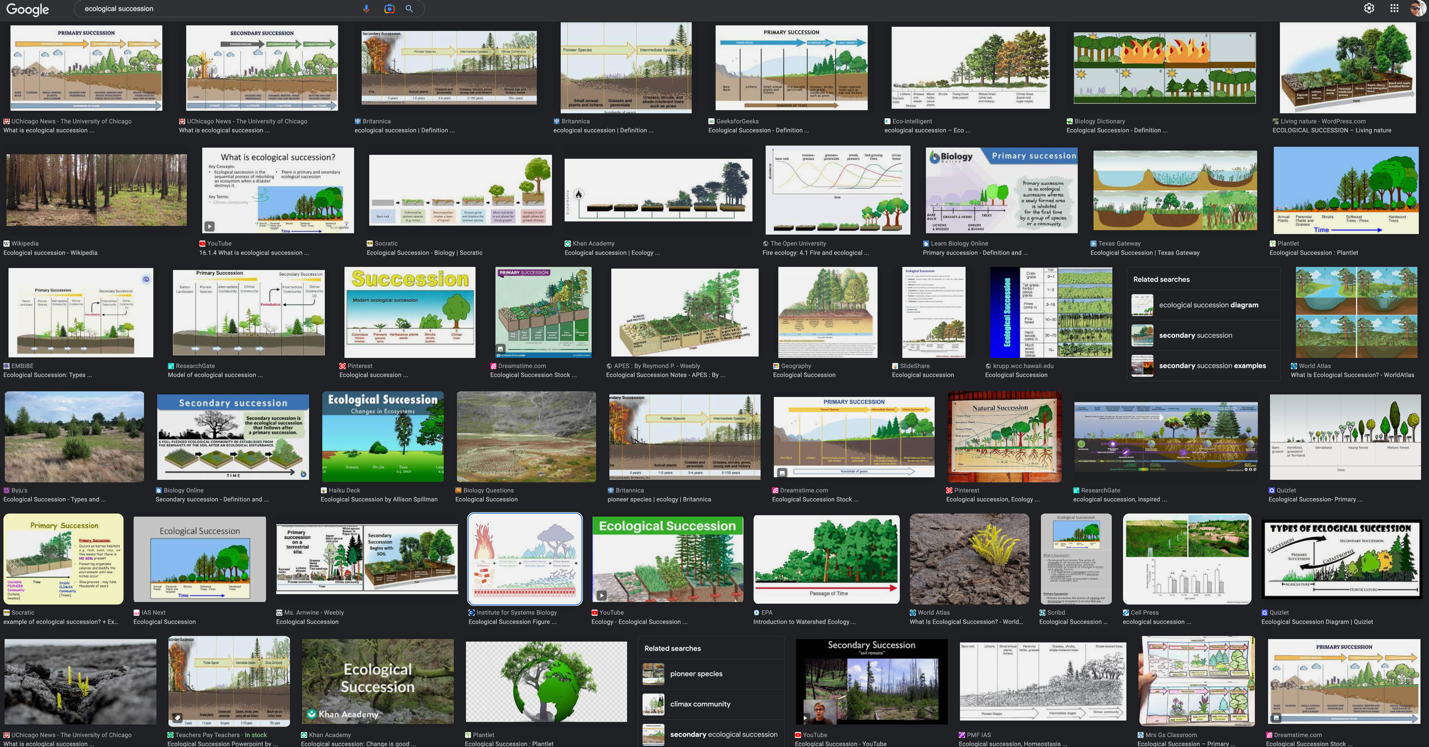
A.


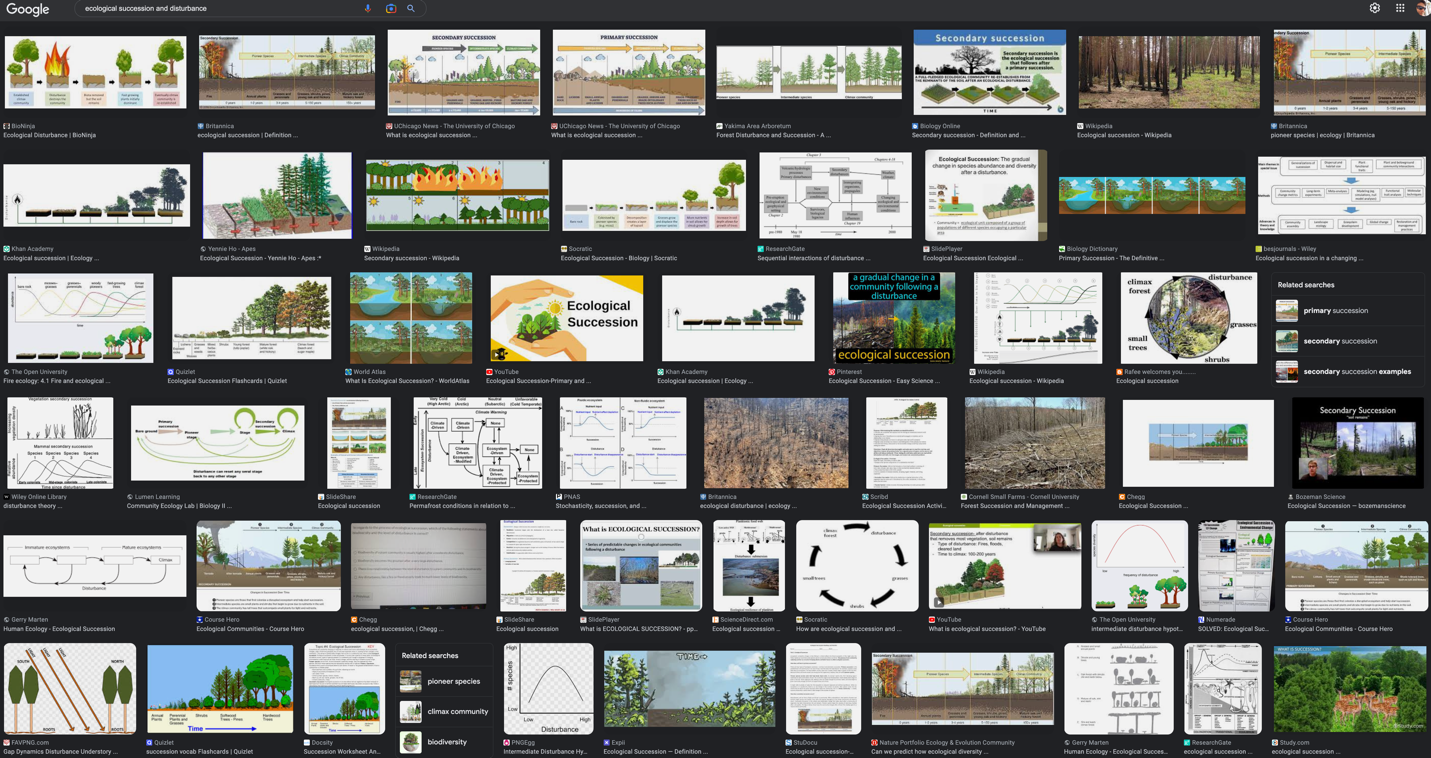
B.
